# Retinopathy Signs Improved Prediction and Reclassification of Cardiovascular Disease Risk in Diabetes: A prospective cohort study

**DOI:** 10.1038/srep41492

**Published:** 2017-02-02

**Authors:** Henrietta Ho, Carol Y. Cheung, Charumathi Sabanayagam, Wanfen Yip, Mohammad Kamran Ikram, Peng Guan Ong, Paul Mitchell, Khuan Yew Chow, Ching Yu Cheng, E. Shyong Tai, Tien Yin Wong

**Affiliations:** 1 Singapore Eye Research Institute, Singapore National Eye Center, Duke-NUS Medical School, 168751, Singapore; 2 Chinese University of Hong Kong Eye Centre, Department of Ophthalmology and Visual Sciences, Hong Kong; 3 Centre for Vision Research, University of Sydney, New South Wales 2006, Australia; 4 Health Promotion Board, National Registry of Diseases Office, 168937, Singapore; 5 National University Hospital Singapore, Division of Endocrinology, 119074, Singapore

## Abstract

CVD risk prediction in diabetics is imperfect, as risk models are derived mainly from the general population. We investigate whether the addition of retinopathy and retinal vascular caliber improve CVD prediction beyond established risk factors in persons with diabetes. We recruited participants from the Singapore Malay Eye Study (SiMES, 2004–2006) and Singapore Prospective Study Program (SP2, 2004–2007), diagnosed with diabetes but no known history of CVD at baseline. Retinopathy and retinal vascular (arteriolar and venular) caliber measurements were added to risk prediction models derived from Cox regression model that included established CVD risk factors and serum biomarkers in SiMES, and validated this internally and externally in SP2. We found that the addition of retinal parameters improved discrimination compared to the addition of biochemical markers of estimated glomerular filtration rate (eGFR) and high-sensitivity C-reactive protein (hsCRP). This was even better when the retinal parameters and biomarkers were used in combination (C statistic 0.721 to 0.774, p = 0.013), showing improved discrimination, and overall reclassification (NRI = 17.0%, p = 0.004). External validation was consistent (C-statistics from 0.763 to 0.813, p = 0.045; NRI = 19.11%, p = 0.036). Our findings show that in persons with diabetes, retinopathy and retinal microvascular parameters add significant incremental value in reclassifying CVD risk, beyond established risk factors.

Cardiovascular disease (CVD) is the leading cause of death in persons with diabetes^1^. Although diabetes was once considered a CVD risk equivalent^2^, heterogeneity of such risk is increasingly being recognized^3^, with new guidelines now including risk assessment specifically for diabetic patients^4^. Yet, there remains a pressing need to derive a CVD prediction algorithm in individuals with diabetes as the Framingham risk scores did not perform desirably in this population^5^,^6^, and existing CVD prediction models in persons with diabetes lack accuracy^7^,^8^. Nonetheless, CVD risk estimates have a pivotal role in allowing physicians to more accurately target and titrate preventive measures such as statin therapy, which is especially important in the diabetic population^9^.

Considerable effort has been directed at the search for novel risk factors to refine global risk prediction^10^,^11^. In particular, high sensitivity C-reactive protein (hsCRP), associated with microvascular complications of diabetes, has consistently demonstrated the ability to add prognostic value to the Framingham Risk score^12^,^13^. Likewise, estimated glomerular filtration rate (eGFR), reflecting the presence of nephropathy, a major microvascular complication persons with diabetes, is shown to be an independent risk factor for CVD outcomes and all-cause mortality^14^,^15^. Thus, eGFR and hsCRP, are quantifiable serum biomarkers that may monitored in conjunction with traditional laboratory markers of CVD during follow-up review for diabetes.

Retinopathy is a common, specific microvascular sign in persons with diabetes and is usually detected from retinal (fundus) photographs during routine screening. The presence of retinopathy in persons with diabetes is known to be associated with increased CVD risk^16^,^17^. Besides these qualitative markers of microvascular pathology, new indicators of microvascular damage such as changes to retinal vascular caliber can now be measured from the same retinal photographs using computer-assisted programs^18^,^19^. Studies now show that these measures of microvascular damage are associated with CVD not only in the general population^20^,^21^, but also in cohorts with diabetes^19^,^22^. This suggests that retinal measures captured from retinal photographs, reflecting generalized microvascular disease, may be potentially useful in further refining CVD risk in persons with diabetes.

In this study, we evaluated the impact of adding retinal signs to traditional CVD risk factors, and a CVD risk prediction algorithm that included retinopathy and retinal vascular caliber assessed from retinal photographs. We also explored the value of additional serum biomarkers of eGFR and hsCRP in CVD prediction. We then examined the robustness of this new prediction algorithm in an independent cohort with diabetes.

## Results

The baseline demographic characteristics of SiMES and SP2 cohorts are shown in Table 1. The mean follow-up was 6.1 years (range: 0.2–7.1, median: 6.3) for SiMES, and 5.78 years (0.29–7.18, 6.06) for SP2. Participants in the SiMES cohort were older, had more women and hypertensive subjects compared with SP2. Participants in SiMES also had higher BMI, systolic blood pressure, total cholesterol and LDL, creatinine level, HbA1c level, and lower eGFR. More participants had retinopathy in the SiMES cohort. However, there was no significant difference in CRAE and CRVE or hsCRP levels between the two cohorts.

During follow-up, 86 cases (12.1%) of CVD occurred in SiMES, of which 29 were MI, 19 were CVD death, 22 were strokes and 16 had mixed etiologies. Of the 31 cases (7.3%) of incident CVD in SP2, 13 were MI, 4 were CVD death, 7 were strokes and 7 had mixed etiologies. Between the two cohorts, there was no difference in incident MI (p = 0.298) or incident stroke (p = 0.187). However, there were significantly more CVD events in SiMES compared with SP2 (p = 0.01).

Table 2 shows that in Model 1, SiMES participants were older, with higher systolic blood pressure and higher HbA1c level were more likely to develop incident CVD. The predictive discrimination for incidence of CVD event of this model was modest, with a C statistic of 0.721. Both a lower GFR and higher hsCRP were significantly associated with increased risk of incident CVD in Model 2. Serum biomarkers improved predictive discrimination of incident CVD, with an increase in the C statistic to 0.747 (p = 0.056). When retinal measures were included in Model 3, discrimination was even better, with an increase in C statistics to 0.751 (p = 0.011). In both Models 2 and 3, the difference compared to Model 1 was not statistically significant (p = 0.0556 and 0.0542, respectively).

The combination of hsCRP, eGFR and the presence of retinopathy in Model 4, significantly improved risk discrimination, with a C statistic of 0.762 (p = 0.011). Model 5 similarly revealed that lower eGFR), higher hsCRP, lower CRAE and the presence of retinopathy are predictors of incident CVD. Model 5 showed a gain in indices of model fit and discrimination, with a statistically significant increase in the C-statistic to 0.774 compared with Model 1 (5.3%, p = 0.003) (ESM Fig. 3).

The Hosmer-Lemeshow χ^2^ p values were p = 0.91 for the Model 1, p = 0.217 for Model 2, p = 0.633 for Model 3, p = 0.677 for Model 4, and p = 0.402 for Model 5, indicating that none of the models had a significant lack of fit.

Table 3 shows the reclassification of CVD risk of Model 1 compared to Model 5. Among individuals with incident CVD event, 12 (14%) participants were reclassified to a higher risk category and 9 (10.5%) participants to a lower risk category (NRI = 3.49%; p = 0.512). In individuals with no incident CVD, the newer model reclassified 62 (10%) participants to a higher risk category and 146 (23.4%) to a lower risk category (NRI = 13.5%, p < 0.001). The overall improvement in net reclassification with the new model was statistically significant (NRI = 17%, p = 0.004).

### Internal Validation

The reclassification table by cross validation within the SiMES cohort is shown in Supplementary Table S1. In subjects with incident CVD, 14 (16.3%) subjects were reclassified to a higher risk category and 11 (12.8%) to a lower risk category. In subjects without incident CVD event, 67 (10.8%) were reclassified to a higher risk, and 148 (23.8%) to a lower risk. The new model performed consistently, with a downward reclassification of subjects with no incident CVD event (NRI 13%; p < 0.001). As a whole, the new model yielded an NRI of 16.5% (p = 0.009), which indicates a better risk stratification of the new model.

### External Validation in SP2

Variables selected in the SiMES refit model (Model 5) and the risk of incident CVD in SP2 was reviewed (Supplementary Table S2). Hazard ratios were largely in the same direction and of similar magnitudes in both cohorts for the Model 1. The new model showed that the presence of retinopathy (HR 0.98, 95% CI, 0.42–2.28) was not associated with incident CVD in SP2. Surprisingly, an increase in venular caliber instead, was significantly associated with CVD risk (HR 2.15, 95% CI, 1.38–3.32) unlike the SiMES cohort. Nevertheless, the C statistics for the new model increased from 0.763 to 0.813 (p = 0.045), representing a significant improvement in predictive discrimination compared to Model 1 in SP2. The Hosmer-Lemeshow χ^2^ p values were p = 0.80 and p = 0.30 for Models 1 and 5, respectively), demonstrating no evidence of poor fit.

For individuals who remained free of CVD, the NRI was statistically significant at 22.3% (p < 0.001), demonstrating an improved identification of truly low-risk patients with additional of retinal parameters. Serum biomarkers and retinal microvascular measures significantly improved CVD risk stratification beyond established risk factors in the SP2 cohort (NRI 19.1%; p = 0.036). This finding was similar to our initial analysis in SiMES (Table 4). Ethnicity was not significantly associated with incident CVD in SP2.

## Discussion

In this prospective population-based study, we characterized CVD risk models with the addition of biochemical markers of hsCRP and eGFR, as well as retinal markers of retinopathy and retinal vascular caliber measured from retinal photographs, then assessed the performance of the models with measures of discrimination, calibration and reclassification. The addition of retinal and serum biomarkers markers significantly improved the predictive value of models compared to traditional risk factors alone, with the addition of retinal parameters proving to be stronger predictors of CVD compared with serum biomarkers of hsCRP and eGFR. When used in combination, both predictive discrimination and reclassification of CVD event were significantly better. Our risk model showed good robustness in both internal and external validation.

To our knowledge, our study is the first to assess the incremental value of retinal parameters to established risk factors for CVD risk prediction and stratification in patients with diabetes. Large epidemiology studies have shown that retinal measures such as arteriolar narrowing and venular widening, are associated with all-cause mortality and incident CVD^16^,^17^,^18^,^20^,^21^,^22^,^23^, therefore changes in retinal vasculature may reflect similar changes in the systemic peripheral and cerebrovascular circulation, providing additional insights into the structure and function of small vessels that are important in the development of CVD^24^. Microcirculation changes in diabetes occur as a result of impaired hemodynamic autoregulation^25^, endothelial dysfunction and defects of metabolism^26^, which incites a inflammatory response^27^. This suggests that retinopathy and retinal microvascular changes are sensitive markers of microvascular dysfunction related to CVD in patients with diabetes. Our current study thus underscored the relationship between retinal measures and incident CVD not only in general population but also in persons with diabetes. We further extend this knowledge by showing that the inclusion of these signs in a CVD risk model demonstrates significant improvement in predictive discrimination and reclassification.

We also demonstrated that a “multiple marker” approach might be better than traditional risk scores for CVD prediction in patients with diabetes. Previous studies that incorporated novel markers (e.g. B-type natriuretic peptide, CRP) into conventional risk models^11^,^12^,^28^, used alone or in tandem with other markers, showed modest change in the C statistic, as they were already strongly correlated with established risk factors, and therefore could not substantially improve risk prediction^11^,^29^. Moreover, previous studies that reviewed the performance of CVD risk scores in persons with diabetes lack complete measures of predictive incremental performance, consisting of discrimination, calibration and model fit^30^. The unique advantage of retinal parameters over other biomarkers in risk prediction is that the retinal vasculature directly reflects microvascular damage and are therefore discrimanatory, whereas other factors are not as specific^31^. While the addition of novel serum biomarkers to established risk factors led to minimal change in the reclassification of cases, our model successfully moved individuals with no incident CVD event to a lower risk category. Better discrimination of CVD risk will empower physicians with the improved ability to administer appropriate treatment, and therefore reduce unnecessary intensification of therapy in patients who are at lower risk of CVD. The utility of our model with retinal parameters in clinical practice is indeed highly feasible, and merits consideration. Many patients with diabetes undergo retinal photography as part of routine yearly eye screening. Data from photographs can be incorporated into risk prediction models without much additional cost. Future studies to assess the acceptability to patients in clinical settings would be insightful.

Strengths of our study include two independent population-based samples, and masked standardized retinal assessment. External validation was performed in an independent population, which proved the accuracy and generalizability of our model^29^,^31^. We used C statistic and NRI, both shown to be more clinically relevant than traditional odds ratio in risk prediction. Moreover, we used NRI to access the effects of the inclusion of the retinal microvascular measures into the models in this study. However, NRI has its limitations; for example, NRI does not provide information about the performances of the models and it weighs reclassifications indiscriminately for three or more risk categories^32^. The incremental and clinical value of the retinal microvascular measures should be interpreted within these limitations and further confirmed in other studies. Limitations include relatively small increase in C statistics, indicating minimal change in overall discrimination, and possibly insufficient clinically meaningful improvement for our model to be adopted as a screening tool. However, the ease of obtaining retinal data in a quick and noninvasive manner may still warrant consideration for future application of these novel markers. Our study had a relatively short follow-up period, and as we only included subjects with diabetes in the current analysis, the sample size is relative small for a population based study. After refitting in SP2, most of the predicting factors from SiMES were insignificant, likely due to the sample size and event rates being insufficient to detect significance in the SP2 cohort. Furthermore, the differences in the strength of associations between risk factors were not taken into account when formulating the prediction models in SP2. Indeed, refitting and recalibration of a model is especially difficult in a heterogenous population. Overfitting in the models of SP2 is also a limitation in out study. Further validation of our model in other cohorts with larger sample sizes will be required. Despite having multiple ethnic groups within SP2, generalization of our findings across ethnic groups may be limited due to small numbers of incident CVD, and further studies are required to recalibrate these functions to fit other populations.

In conclusion, we demonstrated that an assessment of retinopathy and retinal microvascular measures captured from retinal photographs provides prognostic information on risk of CVD and significantly improves CVD risk stratification when incorporated into established risk models in persons with diabetes. The translation of retinal photography as a useful tool to improve the management of diabetes in clinical practice is promising but requires further evaluation.

## Methods

### Study populations

Data from two independent population-based studies was used. Individual data was obtained from the Singapore Malay Eye Study (SiMES, 2004–2006) as the primary cohort to develop the CVD prediction model, which was then validated in the Singapore Prospective Study Program (SP2, 2004–2007). Study populations and methodology for both cohorts have been previously reported^33^,^34^. Of the 3280 eligible individuals from SiMES^33^ selected by an age-stratified random sampling method, 709 persons with diabetes were included in Supplementary Figure S1. 425 persons with diabetes were included from 7744 randomly recruited participants across four cross-sectional studies in SP2^34^ (see Supplementary Figure S2).

The methodology for SiMES and SP2 were similar except that non-fasting blood samples were collected in SiMES and fasting samples were collected in SP2. Written, informed consent was obtained from each participant and both studies adhered to the Declaration of Helsinki. Ethical approval was obtained from the Institutional Review Board at the Singapore Eye Research Institute (SiMES) and the National University of Singapore and Singapore General Hospital (SP2).

### Diagnosis of diabetes

Venous blood samples were analyzed at the National University Hospital Reference Laboratory for biochemical testing. In SiMES, we diagnosed diabetes if participants reported physician diagnosed diabetes or use of diabetic medication, if random glucose was ≥11.1 mmol/L or glycosylated hemoglobin (HbA1c) level ≥6.5%. In SP2, diabetes was diagnosed if participants reported physician diagnosed diabetes or use of diabetes medication, or if fasting plasma glucose was ≥7 mmol/L, and HbA1C> = 6.5%.

### Assessment of established risk factors

Biochemical testing of serum total cholesterol, high-density lipoprotein cholesterol (HDL), low-density lipoprotein cholesterol (LDL), HbA1c, hsCRP and eGFR were obtained from each participant. Current smokers were defined as smoking 1 cigarette or more per day (ie, current versus past/never). Measurements of body mass index (BMI) and blood pressure have previously been reported in detail^33^,^34^. eGFR was estimated from serum creatinine using the recently developed Chronic Kidney Disease Epidemiology Collaboration (CKD-EPI) equation^35^,^36^.

### Retinal Photography

Digital fundus photography was taken with a 45° digital retinal camera (Canon CR-DGi with a 10D SLR digital camera back; Canon, Tokyo, Japan) after pupil dilation using tropicamide 1% and phenylephrine hydrochloride 2.5%. Two retinal images of each eye were obtained, one centered on the optic disc and one centered at the fovea. The spatial resolution of each image was 3072 by 2048 pixels and the images were stored without compression before analysis.

### Retinopathy and Retinal Microvasculature Assessment

Digital retinal photographs were analyzed for retinopathy signs by trained graders based on a standardized protocol. Retinopathy signs (microaneurysms, hemorrhages, or exudates) were deemed present if the retinopathy score (a scale modified from the Airlie House classification system) was at level ≥15^37^.

A semi-automated computer imaging program, the Interactive Vessel Analysis [IVAN (University of Wisconsin, Madison, WI)], was used to measure retinal vascular width within the area of 0.5 to 1.0 disc diameters away from the disc margin. The retinal arteriolar and venular calibers were summarized as central retinal artery equivalent (CRAE) and central retinal vein equivalent (CRVE) respectively^38^. The reproducibility of retinal vascular caliber measurements has been previously reported^38^.

### Assessment of incident CVD

All Singaporean residents have a unique identifier that is linked with the national disease and death registries. We defined incident CVD events as myocardial infarction (MI), stroke or CVD mortality that occurred between baseline examination and 31^st^ December 2011. Incident MI was diagnosed and coded with International Classification of Diseases 9^th^ Revision (International Classification of Diseases-9 [ICD]) 410 in all hospitals, through linkage with the Singapore Myocardial Infarction Registry (SMIR), a nation-wide registry under the purview of the National Registry of Diseases Office (NRDO). Incident MI included [1] definitive or clinical MI; and [2] death cases signed up by pathologists or physicians as MI, with or without necropsy done. Incident stroke data was obtained from the Singapore Stroke Registry gathered from Mediclaims and from public hospital electronic medical records using ICD-9 codes 430 to 433 and 436 to 437, while excluding 432.0, 432.1 and 435 (extradural hemorrhage, subdural hemorrhage, transient cerebral ischemia respectively). All-cause mortality and CVD mortality were ascertained by linkage with the Registry of Births and Deaths. All data collected for the registries were validated and properly anonymized before analysis.

### Statistics

Descriptive data were presented as mean (standard deviation [SD]) for continuous variables or number (percentages) of participants for categorical variables. Cox proportional hazard regression was used to compute hazard ratios (HRs) with 95% confidence interval (CI) for the associations between covariates and CVD outcomes. Proportional hazard assumption was confirmed for all predictors with Schoenfeld’s residuals. Potential predictors of CVD were evaluated with four models, retaining significant variables (p < 0.05) at each stage. Variables were considered as candidates for the model if they were found to be significant at alpha <0.10 by univariate analysis. Variables that remained significant at alpha <0.05 were retained, as well as those that were identified as confounders. Although some confounders were not initially selected due to nonsignificance (such as smoking and cholesterol), as important factors in the Framingham risk calculations, they were included as they may be important contributors to the risk prediction in the presence of the other significant variables. Our basic model (Model 1) included traditional CVD risk factors^39^. Serum CVD biomarkers (eGFR and hsCRP) were added to Model 1 (Model 2). Retinal signs were added in Model 3, and separately in Model 4 (Model 2 and retinopathy) and Model 5 (Model 4 and retinal vascular calibers). The variables were retained at each stage so as to build up the nested model structure eventually.

Prognostic comparisons between models were done using Harrell’s concordance C-statistic^40^. Effects of the inclusion of the retinal microvascular measures into the models were assessed using the Net Reclassification Improvement (NRI)^41^.

Because there are no recommended risk thresholds for diabetes in clinical practice, the predicted probabilities of CVD event were treated as a continuous variable and the approximate tertile cutoffs were used as threshold points. Based on these 2 cut-off points, subjects were stratified into low, intermediate, and high-risk categories. The predicted probability for the incident CVD outcome were regenerated using both retinal measures and established risk factors, and the subjects were re-assigned to low, intermediate or high risk categories.

Hosmer-Lemeshow chi-square statistic was calculated for the models to test goodness-of-fit. P < 0.05 represents a significant difference between the expected and observed event rates, indicating that the model is not well calibrated.

### Validation of the prediction model

The prediction models were validated using internal validation and external validation.

In order to avoid over-fitting and optimism in model performance, the final model for CVD events among diabetic individuals was internally cross validated. Internal validation is a measure of how well a model is able to predict the outcome for new observations that were not used in developing the model. Ten-fold cross validation was implemented by randomly generating 10 test samples using 10-fold cross-validation within the SiMES cohort. The data was randomly spilt into 10 mutually exclusive subsets (S1–S10) of equal sizes containing approximately the same number of events. From this, we generated 10 test samples leaving out subset S1 and so on. NRI was then calculated using the statistics from all 10 subsets.

To externally validate the models in the SP2, the strength of associations between risk factors identified and CVD were first evaluated for similarities between the SP2 and SiMES cohorts. The coefficients (hazard ratios) for all variables selected in the SiMES model were refitted to the SP2 cohort, even though some of the risk factors in SiMES did not show significance in SP2. As our aim was to validate Model 5 from SiMES in another cohort, we kept all the variables to remain consistent with the SiMES model. Hosmer-Lemeshow χ^2^ p values were calculated to calibrate Model 1 and Model 5 in the SP2 cohort. The coefficients from the SiMES models were then recalibrated to the baseline survival of SP2, in order to perform model test similar to those in SiMES. As we expected there to be ethnic differences between groups, ethnicity first reviewed as an independent variable in univariate analysis. However, it was found not to be significant in SP2, so we chose to omit ethnicity to remain consistent with the SiMES model.

We regarded P values of <0.05 from 2-sided tests to indicate statistical significance. All statistical analyses were performed using the Stata Statistical computer package (STATA Statistical Software, Version 12, Statacorp, College Station, Texas, USA).

## Additional Information

**How to cite this article:** Ho, H. *et al*. Retinopathy Signs Improved Prediction and Reclassification of Cardiovascular Disease Risk in Diabetes: A prospective cohort study. *Sci. Rep.* **7**, 41492; doi: 10.1038/srep41492 (2017).

**Publisher's note:** Springer Nature remains neutral with regard to jurisdictional claims in published maps and institutional affiliations.

## Supplementary Material

Supplementary Dataset 1

## Figures and Tables

**Table 1:** Comparison of Baseline Characteristics between Singapore Malay Eye Study (SiMES) and Singapore Prospective Study-2 (SP2) participants.

Characteristics	SiMES	SP2	p-value
	(n = 709)	(n = 425)	
Age, mean (SD), years	60.2 (9.71)	56.90 (9.61)	<0.001
Ethnicity, n (%)			<0.001
Chinese	0 (0.00)	177 (41.65)	
Malay	709 (100.00)	107 (25.18)	
Indian	0 (0.00)	141 (33.18)	
Gender, male	319 (44.99)	229 (53.88)	0.004
Current smoking, yes (%)	119 (16.78)	54 (12.71)	0.050
Hypertension, yes (%)	577 (81.38)	270 (63.53)	<0.001
Systolic BP, mean (SD), mmHg	152.86 (22.00)	142.86 (21.68)	<0.001
Diastolic BP, mean (SD), mmHg	80.55 (10.78)	80.60 (10.53)	0.946
Total Cholesterol, mean (SD), mmol/L	5.63 (1.22)	5.28 (1.09)	<0.001
HDL-cholesterol, mean (SD), mmol/L	1.29 (0.30)	1.30 (0.30)	0.569
LDL-cholesterol, mean (SD), mmol/L	3.48 (0.99)	3.23 (0.96)	<0.001
HbA1c, % (mmol/mol)	8.06 (65)	7.64 (60)	<0.001
Duration of diabetes, mean (SD), years	5.18 (7.97)	4.54 (8.06)	0.182
Hypoglycemic medication, yes (%)	316 (44.57)	191 (42.07)	0.402
Anti-hypertensive medication, yes (%)	236 (33.29)	161 (37.88)	0.116
Anti-hyperlipidaemia medication, yes (%)	156 (22.23)	116 (27.29)	0.043
eGFR, mean (SD), mL/min/1.73 m^2^	73.60 (22.23)	81.74 (18.18)	<0.001
hsCRP, mean (SD), mg/L	4.19 (5.89)	4.28 (8.40)	0.843

p-value based on chi-squared test, or independent-samples t-test, where appropriate.

SiMES: Singapore Malay Eye Study; SP2: Singapore Prospective Study-2; BMI: body mass index; Hba1c: glycated haemoglobin (A1c); HDL: high density lipoprotein; LDL: low density lipoprotein; eGFR: estimated glomerular filtration rate; HsCRP: high-sensitivity C-reactive protein.

**Table 2:** Relation of establish risk factors, serum biomarkers and retinal microvascular measures and the risk of cardiovascular events for the SiMES cohort.

	Model 1	Model 2	Model 3	Model 4	Model 5
	HR (95% CI)	HR (95% CI)	HR (95% CI)	HR (95% CI)	HR (95% CI)
Age (years)	**1**.**06** (**1**.**04**, **1**.**09**)	**1**.**04** (**1**.**01**, **1**.**07**)	**1**.**06** (**1**.**03**, **1**.**09**)	**1**.**06** (**1**.**03**, **1**.**07**)	**1**.**06** (**1**.**03**, **1**.**09**)
Gender, male	0.89 (0.53, 1.49)	0.85 (0.53, 1.37)	0.86 (0.51, 1.46)	0.88 (0.52, 2.48)	0.82 (0.50, 1.34)
Total cholesterol (mmol/L)	1.16 (0.97, 1.38)	1.14 (0.96, 1.34)	**1**.**17** (**0**.**98**, **1**.**40**)	1.16 (0.98, 1.37)	**1**.**19** (**1**.**00**, **1**.**41**)
HDL cholesterol (mmol/L)	0.56 (0.25, 1.25)	0.62 (0.28, 1.36)	0.59 (0.26, 1.32)	0.69 (0.32, 1.53)	0.71 (0.33, 1.55)
Current Smoking	1.42 (0.79, 2.55)	1.59 (0.88, 2.90)	1.51 (0.83, 2.75)	1.54 (0.85, 2.80)	1.69 (0.91, 3.12)
HbA1c (%)	**1**.**14** (**1**.**02**, **1**.**26**)	**1**.**16** (**1**.**04**, **1**.**28**)	**1**.**11** (**0**.**99**, **1**.**24**)	**1**.**13** (**1**.**01**, **1**.**26**)	**1**.**12** (**1**.**01**, **1**.**26**)
Diabetic duration (years)	1.01 (0.99, 1.04)	1.01 (0.99, 1.04)	1.01 (0.98, 1.03)	1.01 (0.98, 1.03)	1.00 (0.98, 1.03)
Hypoglycemic medication	0.91 (0.53, 1.55)	0.93 (0.57, 1.50)	0.85 (0.49, 1.48)	0.86 (0.50, 1.47)	0.91 (0.56, 1.50)
Systolic blood pressure (mmHg)	1.01 (1.00, 1.02)	1.01 (1.00, 1.02)	1.00 (0.99, 1.01)	1.00 (0.99, 1.01)	1.00 (0.99, 1.01)
Anti-hypertensive medication	0.75 (0.42, 1.35)	0.69 (0.38, 1.25)	0.81 (0.45, 1.46)	0.69 (0.38, 1.24)	0.73 (0.40, 1.33)
Anti-hyperlipidaemia medication	1.20 (0.63, 2.28)	1.13 (0.59, 2.14)	1.25 (0.65, 2.38)	1.28 (0.67, 2.45)	1.29 (0.67, 2.51)
hsCRP (mg/L)		**1**.**41** (**1**.**10**, **1**.**79**)		**1**.**43** (**1**.**12**, **1**.**82**)	**1**.**41** (**1**.**10**, **1**.**81**)
eGFR (mL/min/1.73 m^2^)		**1**.**02** (**1**.**01**, **1**.**03**)		**1**.**01** (**1**.**01**, **1**.**03**)	**1**.**01** (**1**.**01**, **1**.**02**)
Presence of retinopathy, yes			**2**.**13** (**1**.**29**, **3**.**53**)	**1**.**94** (**1**.**17**, **3**.**21**)	**2**.**04** (**1**.**27**, **3**.**28**)
Retinal arteriolar caliber^a^ (μm)			**1**.**37** (**1**.**06**, **1**.**77**)		**1**.**35** (**1**.**04**, **1**.**83**)
Retinal venular caliber^b^ (μm)			1.17 (0.00, 4.26)		1.19 (0.92, 1.53)

^a^per standard deviation decrease in arteriolar caliber.

^b^per standard deviation increase in venular caliber.

HR: hazard ratio: CI: confidence interval; HDL: high density lipoprotein; LDL: low density lipoprotein; Hba1c: glycated haemoglobin (A1c); hsCRP: high-sensitive C reactive protein; eGFR: estimated glomerular filtration rate. Model 1: Established risk factors. Model 2: Established risk factors + serum biomarkers (). Model 3: Established risk factors + retinal measures (presence of retinopathy, retinal arteriolar caliber and retinal venular caliber). Model 4: Established risk factors + serum biomarkers + presence of retinopathy. Model 5: Established risk factors + serum biomarkers + retinal measures.

**Table 3:** Risk of Cardiovascular Event Predicted by Model of Established Risk Factors and New Model* in the Diabetic Cohort from the Singapore Malay Eye Study.

	^+^‘New’ Risk Category				Reclassified to Higher risk	Reclassified to Lower risk	NRI, %	P-value
	Low	Intermediate	High	Total				
Subjects with incident CVD event								
*Established Risk Category					12	9	3.49	0.512
Low	7	1	0	8				
Intermediate	2	10	11	23				
High	0	7	48	55				
Total	9	18	59	86				
Subjects with no incident CVD event								
*Established Risk Category					62	146	13.48	<0.001
Low	179	26	5	210				
Intermediate	89	104	31	224				
High	1	56	132	189				
Total	269	186	168	623				
NRI, %							16.97	0.004

^+^Established risk factors include age (years), gender (male, female), HbA1c, systolic blood pressure (mmHg), total cholesterol (mmol/L), HDL cholesterol (mmol/L), current smoking (yes, no), diabetic medication (yes, no) and duration of diabetes (years).

*New risk factors include established risk factors, serum biomarkers (eGFR and hsCRP) and retinal microvascular parameters (retinal arteriolar caliber, retinal venular caliber, retinal vascular fractal dimension and presence of retinopathy).

NRI: Net reclassification improvement.

Risk Categories %.

Low 0− < 6.35.

Intermediate 6.35–13.2.

High >13.2.

**Table 4:** External-Validation for Risk of Cardiovascular Event Risk Prediction in the Singapore Prospective Study 2.

	*‘New’ Risk Category				Reclassified to Higher risk	Reclassified to Lower risk	NRI, %	P-value
	Low	Intermediate	High	Total				
Subjects with incident CVD event								
^+^Established Risk Category					3	4	−3.23	0.7055
Low	1	1	1	3				
Intermediate	3	5	1	9				
High	0	1	18	19				
Total	4	7	20	31				
Subjects with no incident CVD event								
^+^Established Risk Category					37	125	22.34	<0.001
Low	126	10	4	140				
Intermediate	70	37	23	130				
High	29	26	69	124				
Total	225	73	96	394				
NRI, %							19.11	0.036

^+^Established risk factors include age (years), gender (male, female), HbA1c, systolic blood pressure (mmHg), total cholesterol (mmol/L), HDL cholesterol (mmol/L), current smoking (yes, no), diabetic medication (yes, no) and duration of diabetes (years).

*New risk factors include established risk factors, serum biomarkers (eGFR and hsCRP) and retinal microvascular parameters (retinal arteriolar caliber, retinal venular caliber, retinal vascular fractal dimension and presence of retinopathy).

NRI: Net reclassification improvement.

Risk Categories %.

Low 0 − < 3.7.

Intermediate 3.7–7.1.

High >7.1.
