# Supplementary material for: Retinopathy Signs Improved Prediction and Reclassification of Cardiovascular Disease Risk in Diabetes: A prospective cohort study
Source: Sci Rep. 2017 Feb 2;7:41492. doi: 10.1038/srep41492 (PMC5288652; doi:10.1038/srep41492)

**Retinopathy Signs Improved Prediction and Reclassification of Cardiovascular Disease Risk in Diabetes**

***A prospective cohort study***

Henrietta Ho^1,+^, Carol Y Cheung^1,2,+^, Charumathi Sabanayagam^1^, Wanfen Yip^1^, Mohammad Kamran Ikram^1^, Peng Guan Ong^1^, Paul Mitchell^3^, Khuan Yew Chow^4^, Ching Yu Cheng^1^, E Shyong Tai^5^, Tien Yin Wong^1,+^,*

^1^Singapore Eye Research Institute, Singapore National Eye Center, Singapore 168751

^2^Chinese University of Hong Kong Eye Centre, Department of Ophthalmology and Visual Sciences, Hong Kong

^3^Centre for Vision Research, University of Sydney, New South Wales 2006, Australia

^4^Health Promotion Board, National Registry of Diseases Office, Singapore 168937

^5^National University Hospital Singapore, Division of Endocrinology, Singapore 119074

*These authors contributed equally to this work

Correspondence and requests for materials should be addressed to Prof Tien Y Wong ([wong.tien.yin@snec.com.sg](mailto:wong.tien.yin@snec.com.sg))

**Supplementary Information**

1. Supplementary Table S1: Internal 10-fold Cross-Validation for Risk of Cardiovascular Event Risk Prediction with Model of Established Risk Factors and New Model from the Singapore Malay Eye Study
2. Supplementary Table S2: Relation of establish risk factors and retinal microvascular measures to risk of cardiovascular events for the SP2 cohort
3. Supplementary Figure S1: Recruitment of individuals from the Singapore Malay Eye Study (SiMES)
4. Supplementary Figure S2: Recruitment of individuals from the Singapore Prospective Study Program (SP2)

**Supplementary Table S1: Internal 10-fold Cross-Validation for Risk of Cardiovascular Event Risk Prediction with Model of Established Risk Factors and New Model* from the Singapore Malay Eye Study**

|  | ^*^‘New’ Risk Category | | | | Reclassified to Higher risk | Reclassified to Lower risk | NRI, % | P-value |
| --- | --- | --- | --- | --- | --- | --- | --- | --- |
|  | Low | Intermediate | High | Total |
| Subjects with incident CVD event |  |  |  |  |  |  |  |  |
| ^+^Established Risk Category |  |  |  |  | 14 | 11 | 3.49 | 0.549 |
| Low | 9 | 5 | 0 | 15 |  |  |  |  |
| Intermediate | 4 | 9 | 8 | 21 |  |  |  |  |
| High | 0 | 7 | 43 | 50 |  |  |  |  |
| Total | 13 | 21 | 52 | 86 |  |  |  |  |
| Subjects with no incident CVD event |  |  |  |  |  |  |  |  |
| ^+^Established Risk Category |  |  |  |  | 67 | 148 | 13.0 | <0.001 |
| Low | 190 | 25 | 7 | 222 |  |  |  |  |
| Intermediate | 90 | 87 | 35 | 212 |  |  |  |  |
| High | 4 | 54 | 131 | 189 |  |  |  |  |
| Total | 284 | 166 | 173 | 623 |  |  |  |  |
| NRI, % |  |  |  |  |  |  | 16.49 | 0.009 |

^+^ Established risk factors include age (years), gender (male, female), HbA1c, systolic blood pressure (mmHg), total cholesterol (mmol/L), HDL cholesterol (mmol/L), current smoking (yes, no), diabetic medication (yes, no) and duration of diabetes (years)

^*^New risk factors include established risk factors, serum biomarkers (eGFR and hsCRP) and retinal microvascular parameters (retinal arteriolar caliber, retinal venular caliber, retinal vascular fractal dimension and presence of retinopathy)

NRI: Net reclassification improvement

| Risk Categories | % |
| --- | --- |
| Low | 0 – <6.8 |
| Intermediate | 6.8 – 13.4 |
| High | >13.4 |
|  |  |

**Supplementary Table S2:** Relation of establish risk factors and retinal microvascular measures to risk of cardiovascular events for the SP2 cohort

|  | **Model 1** | **Model 5** |
| --- | --- | --- |
|  | **HR (95% CI)** | **HR (95% CI)** |
| Age (years) | **1.04 (1.01, 1.09)** | **1.04 (1.01, 1.09)** |
| Gender, male | 0.53 (0.21, 1.29) | 0.48 (0.19, 1.23) |
| Total cholesterol (mmol/L) | 1.09 (0.78, 1.52) | 1.21 (0.78, 1.60) |
| HDL (mmol/L) | 0.21 (0.05, 1.00) | 0.26 (0.06, 1.17) |
| Current smoking | **3.03 (1.28, 7.14)** | 1.69 (0.91, 3.12) |
| HbA1c (%) | 1.19 (0.99, 1.43) | 1.17 (0.97, 1.42) |
| Diabetic duration (years) | 1.02 (0.97, 1.06) | 1.04 (0.98, 1.08) |
| Hypoglycemic medication | 1.48 (0.59, 3.71) | 0.91 (0.56, 1.50) |
| Systolic blood pressure (mmHg) | 1.01 (0.99, 1.03) | 1.01 (0.99, 1.03) |
| Anti-hypertensive medication | 0.39 (0.15, 1.00) | 0.40 (0.15, 1.09) |
| Anti-hyperlipidaemia medication | 0.51 (0.18, 1.41) | 0.62 (0.21, 1.82) |
| hsCRP (mg/L) |  | **1.31 (1.04, 1.64)** |
| eGFR (mL/min/1.73m^2^) |  | 0.99 (0.96, 1.00) |
| Presence of retinopathy, yes |  | 0.98 (0.42, 2.28) |
| Retinal arteriolar caliber^a^ (μm) |  | 1.12 (0.73, 1.71) |
| Retinal venular caliber^b^ (μm) |  | **2.15 (1.38, 3.32)** |

^a^per standard deviation decrease in arteriolar caliber

^b^per standard deviation increase in venular caliber

HR: hazard ratio: CI: confidence interval; HDL: high density lipoprotein; LDL: low density lipoprotein; Hba1c: glycated haemoglobin (A1c); hsCRP: high-sensitive C reactive protein; eGFR: estimated glomerular filtration rate

**Supplementary Figure S1:** Number of subjects considered for inclusion in study of retinal microvascular parameters and CVD risk algorithm, Singapore Malay Eye Study (SiMES)


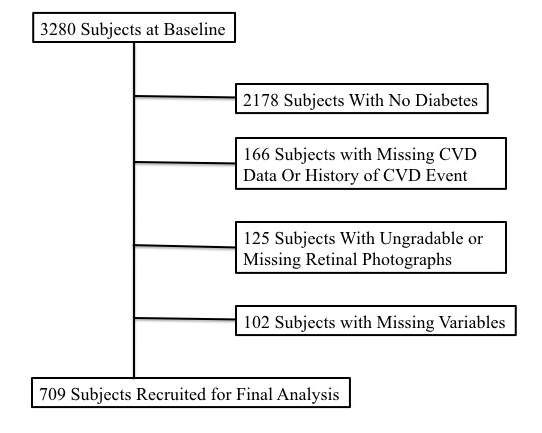


**Supplementary Figure S2:** Number of subjects considered for inclusion in study of retinal microvascular parameters and CVD risk algorithm, Singapore Prospective Study Program (SP2)


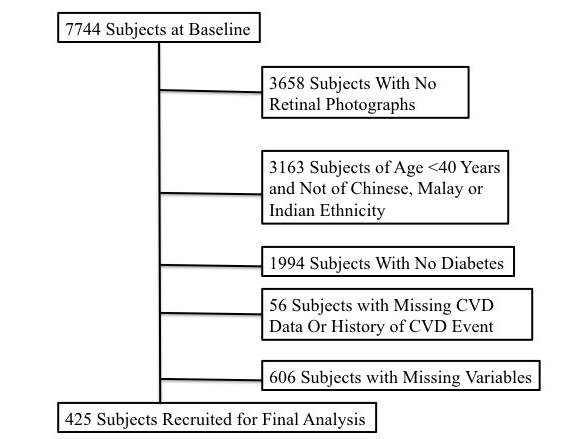

Supplement: Supplementary Dataset 1 [file srep41492-s1.doc]
